# Nodular Hidradenoma With Atypical Features in a Young Patient: A Case Report

**DOI:** 10.7759/cureus.76630

**Published:** 2024-12-30

**Authors:** Ahmad M Tayeb, Ibrahim S Allihibi, Fahad Aljuaid, Khalid Alshareef, Afnan Hasanain, Jaudah Almaghrabi, Latifah Kutbi

**Affiliations:** 1 Dermatology Training Joint Program in Western Region, Ministry of Health, Jeddah, SAU; 2 Department of Dermatology, King Faisal Hospital, Makkah, SAU; 3 College of Medicine, King Saud Bin Abdulaziz University for Health Sciences, Jeddah, SAU; 4 Department of Dermatology, King Faisal Specialist Hospital and Research Centre, Jeddah, SAU; 5 Department of Pathology, Faculty of Medicine, King Abdul-Aziz University Hospital, Jeddah, SAU; 6 Department of Pathology and Laboratory Medicine, King Faisal Specialist Hospital and Research Centre, Jeddah, SAU; 7 Saudi Internal Medicine Residency Training Program, Ministry of Health, Jeddah, SAU

**Keywords:** atypical features, case report, dermatology, histopathology, nodular hidradenoma

## Abstract

Eccrine acrospiromas, also known as hidradenomas, are rare benign tumors that develop from the eccrine sweat glands. Hidradenoma is a multilobular, nonencapsulated, well-circumscribed dermal nodule that may involve the epidermis and extend into the subcutaneous fat. The etiology and prevalence of nodular hidradenoma are not well defined, but it is noted that it can occur spontaneously or traumatically. A 22-year-old, medically healthy male patient presented to the Dermatology clinic with a painless, progressive scalp lesion that had been bleeding on manipulation for the previous three years. On examination, a solitary, non-tender, soft-to-firm, pedunculated red scalp mass with a smooth surface, measuring 2 x 2 x 3 cm, was found over the left occipital area, with mild tenderness on touching. Excision was performed and sent for histopathological examination, which showed an adnexal tumor with features consistent with nodular hidradenomas, such as the tumor involving deep and peripheral margins. In a focal area, the mitotic rate was up to 3/10 HPF (high-power field) and was associated with a focal area of necrosis, which are considered atypical features. The differential diagnosis includes hidradenocarcinoma, renal cell carcinoma, basal cell carcinoma, squamous cell carcinoma, keratoacanthoma, trichoblastoma, trichilemmoma, cystadenoma, syringoma, and sebaceous adenoma.

## Introduction

Nodular hidradenoma is a rare appendageal tumor that occurs in adults and also in children, but is less common. Since it is quite rare, the exact prevalence is poorly documented. It usually appears as a solitary, slow-growing, well-circumscribed, mobile, hard, non-tender cutaneous lesion with a diameter ranging from 0.5 to 12 cm. The appearance of brown, blue, or red discoloration, together with superficial ulceration and serous discharge, can sometimes be mistaken for malignancies. The scalp, neck, trunk, and extremities are the most prevalent sites for nodular hidradenomas. Histopathology is frequently used to confirm the diagnosis [1,2]. The differential diagnosis includes hidradenocarcinoma, renal cell carcinoma, basal cell carcinoma, squamous cell carcinoma, keratoacanthoma, trichoblastoma, trichilemmoma, cystadenoma, syringoma, and sebaceous adenoma [3]. This study aimed to present a case of nodular hidradenoma with atypical features at King Faisal Specialist Hospital in Jeddah.

## Case presentation

**Figure 1:**
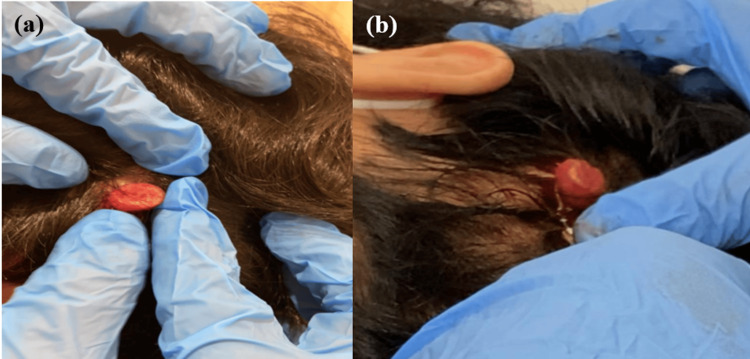
Scalp lesion (a) A solitary non-tender soft to firm pedunculated red scalp mass with a smooth surface measuring 2 x 2 x 3 cm over the left occipital area with mild tenderness with touching; (b) Different view of the scalp lesion

A 22-year-old, medically healthy male patient presented to the Dermatology clinic with a painless, progressive scalp lesion that had been bleeding on manipulation for the previous three years. There was no family history of a similar presentation, and no history of predisposing factors, such as sun or chemical exposure. On examination, a solitary, non-tender, soft-to-firm, pedunculated red scalp mass with a smooth surface, measuring 2 x 2 x 3 cm, was noted over the left occipital area, with mild tenderness on touch (Figures 1a-1b). There was a small erosion in the surrounding area, with no regional lymphadenopathy.

**Figure 2:**
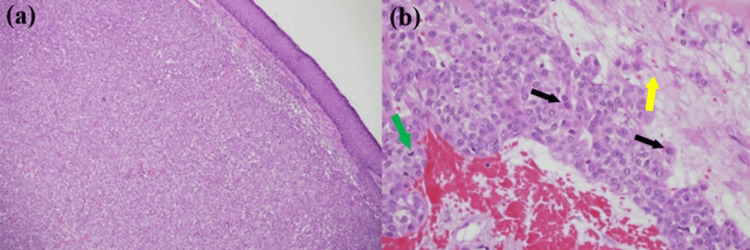
Histologic findings (a) Section shows well-circumscribed but unencapsulated, solid tumor with no overlying connection to the epidermis (hematoxylin and eosin, 40×); (b) Higher power image shows a tumor composed of polyhedral cells (black arrows) with eosinophilic to clear cytoplasm, nuclear atypia, focal area of necrosis (yellow arrow) and some mitotic figure (green arrow) (hematoxylin and eosin, 400×)

A simple excision of the lesion under local anesthesia was performed in the clinic and sent for histopathological examination, which showed an adnexal tumor consistent with nodular hidradenoma with atypical features. The tumor involved deep and peripheral margins. In a focal area, the mitotic rate was up to 3/10 HPF (high-power field) and was associated with a focal area of necrosis. No current guidelines are available; however, the tumor was not invasive, and there were no systemic symptoms or findings during the examination that could indicate a risk of metastasis to other sites. These features are considered atypical. Additionally, there were no histopathological features of hidradenocarcinoma (Figures 2a-2b).

In immunohistochemistry, tumor cells were positive for CK7, CK5/6, pankeratin, P63, and AE/AE3. They were negative for PAX-8, CD34, CEA, MSA, and SMA. Ber-EP4 and Cam5.2 showed focal weak staining. P53 exhibited scattered stained cells, consistent with the wild type. Ki67 was positive in 3% of tumor cells. Epithelial membrane antigen (EMA) was not available. This immunohistochemistry profile is helpful in ruling out the main differential diagnosis, which is metastatic renal cell carcinoma, a clear cell type. Renal cell carcinoma is classically positive for PAX-8 and P63, and negative for CK7, which is the opposite of the immunoexpression in our case. The expression of the other markers also supports the diagnosis of nodular hidradenoma. The patient was referred to plastic surgery for complete excision of the tumor due to the presence of atypia. The patient is following up in our clinic post-operatively, and the lesion has resolved.

## Discussion

Nodular hidradenoma, which has numerous names in the literature, such as eccrine acrospiroma, clear cell hidradenoma, and eccrine spiradenoma, is a benign tumor that arises from the atrichial sweat glands. Based on histologic and histochemical research, Johnson and Helwig [4] developed the name acrospiroma. Histopathology revealed that the tumor matched the cells and structure of the ductal section of sweat glands [1]. The etiology and prevalence of nodular hidradenoma are not well defined, but it is noted that it can occur spontaneously or traumatically. Hernández-Pérez and Cestoni-Parducci [5] found a female predisposition (1.7:1) in a series of patients, with a mean age at presentation of 37.2 years. This tumor typically appears between the fourth and eighth decades of life, with the highest frequency in the sixth decade. According to their findings, the head (30.3%) was the most common site of involvement, followed by the upper limb (25.8%), and finally the trunk (20.2%). Histopathology showed well-confined, strongly delineated lesions from the epidermis, with a clear-cut grenz zone [6]. The two different types of nodular hidradenoma are the apocrine (clear cell hidradenoma) and eccrine (poroid) differentiation types. Clear-cell hidradenoma is the most prevalent type [7,8].

Patients experience discomfort when pressing on the tumor, as well as pruritus or burning around the lesion. Slow-growing, asymptomatic, solitary, mobile, and firm cutaneous nodules are the most prevalent form of these lesions. The skin's swollen surface ranges in color from pink-red to blue-brown or purple, with ulceration on occasion. Local recurrences of these tumors are common, but malignant transformation is extremely uncommon. Histopathologically, they are characterized by well-circumscribed, encapsulated, nodular, solid, or solid-cystic lesions in the dermis. These may be connected to the epidermis, and the dermal epithelial lobules extend into the underlying subcutaneous fat.

When nodular hidradenoma shows atypical histological features, a detailed evaluation is essential to assess the potential for malignant transformation. Although nodular hidradenomas are typically benign, rarely do persistent lesions in the literature show progression to hidradenocarcinoma, which is a rare and aggressive form of sweat gland carcinoma [9]. Some reports in the literature describe cases where nodular hidradenomas show both benign and malignant features, as in our case, which might indicate an early step toward malignancy. However, these cases are rare, and the cause of transformation is not fully understood [10]. It is important for clinicians to stay alert when dealing with lesions that have unusual histological patterns, as they could progress to a more malignant state. Regular monitoring and surgical removal, as done in our case, are the best methods to reduce this risk [11].

Two kinds of epithelial cells make up the solid portions of the tumor. One of the cell types is known as clear cells, which have a tiny, eccentrically situated nucleus, a well-defined cell boundary, and a clear cytoplasm. Another type has a round or oval nucleus with an indefinite cell border, and an eosinophilic, solid or finely granular, or vesicular cytoplasm. Transitional cells, keratinizing cells, and horn pearls are some of the other cells that can be found. Within the solid lobules, tubular lumina resembling eccrine sweat ducts and cystic cavities with homogeneous eosinophilic material can be observed. Solid cell masses and cystic spaces are surrounded by structural connective tissue-stromal support [2]. Oncocytic, epidermoid, and pigmented types, with melanocytes and melanin pigment in cells and macrophages, are some of the other cellular variables [12-14]. Poor circumscription, huge size, solid sheet-like growth pattern, necrosis, vascular and lymphatic invasion, pleomorphism, and a high mitotic rate are all seen in hidradenocarcinoma or malignant hidradenoma histopathology [15].

The most typical appearance seen on dermoscopy is a homogeneous background comprising the whole lesion, with vascular features such as arborizing telangiectasias, linear-irregular vessels, dotted vessels, glomerular vessels, hairpin vessels, telangiectasias, and polymorphous atypical vessels. The histopathological correlation of this homogeneous region is the existence of huge lobulated masses of predominantly polyhedral eosinophilic and clear cells, with various-sized ductal structures and extensive cystic gaps in the upper and mid dermis. Because of the branching vascular pattern with a staghorn structure, these patterns are appreciated [16]. Clinical differential diagnoses include epidermal cysts, cutaneous metastases, basal cell carcinoma, amelanotic melanoma, keratoacanthoma, trichoblastoma, cystadenoma, syringoma, sebaceous adenoma, pilomatricoma, dermatofibroma, neurofibroma, glomus tumor, angioleiomyoma, and leiomyoma. In addition, histopathological differential diagnoses include hidradenocarcinoma, metastatic renal cell carcinoma, basal cell carcinoma with eccrine differentiation, squamous cell carcinoma, trichilemmoma, poroma, and cylindroma [3,16].

## Conclusions

In conclusion, eccrine acrospiromas, or hidradenomas, are rare benign tumors that originate from eccrine sweat glands and are extremely uncommon in becoming malignant. The case highlights a 22-year-old male with a progressive scalp lesion, diagnosed as nodular hidradenoma, characterized by its multilobular and nonencapsulated nature. Despite its benign status, the lesion exhibited atypical features, including a focal area of necrosis and a high mitotic rate. Histopathological examination confirmed the diagnosis and ruled out malignancy. Early diagnosis and appropriate surgical intervention are crucial for such cases. This case underscores the importance of a thorough clinical and histopathological evaluation in managing rare skin tumors effectively.
